# Phosphorylation of syntenin-1 by TBK1 promotes proliferation and migration of non-small cell lung cancer cells

**DOI:** 10.1016/j.jbc.2025.110278

**Published:** 2025-05-22

**Authors:** Lin Gao, Hecun Zou, Guojiao Xie, Xinning Li, Zan Chen

**Affiliations:** Basic Medicine Research and Innovation Center for Novel Target and Therapeutic Intervention, Ministry of Education, College of Pharmacy, Chongqing Medical University, Chongqing, China

**Keywords:** syntenin-1, TBK1, phosphorylation, NSCLC

## Abstract

Syntenin-1 is a promising therapeutic target for cancer, as its inhibitors have shown positive efficacy in preclinical models of various cancer types. Posttranslational modifications, including phosphorylation, play an important role in regulating syntenin-1 activity, but the underlying molecular mechanisms have not been completely understood. To figure out the enzymes that catalyze syntenin-1 modifications, we performed mass spectrometry proteomics analysis of immunoprecipitated syntenin-1 and identified TANK-binding kinase 1 (TBK1) as a binding partner. Using biochemical and cellular assays, we demonstrated that TBK1 directly interacted with syntenin-1 and phosphorylated it at residue S6. ULK1, the reported kinase to catalyze syntenin-1 S6 phosphorylation, was shown in our assays to indirectly trigger syntenin-1 phosphorylation by activating TBK1. We also found that syntenin-1 was upregulated in non-small cell lung cancer (NSCLC) cells, and TBK1-catalyzed syntenin-1 phosphorylation promoted cell growth and metastasis of the NSCLC cell line A549. Transcriptome sequencing revealed that syntenin-1 phosphorylation by TBK1 activated the MAPK signaling pathway. Our study illuminated a new mechanism in which syntenin-1 phosphorylation, regulated by upstream TBK1 signaling, controls NSCLC progression.

Syntenin-1, encoded by SDCBP gene, is a highly conserved protein that acts as an adaptor and scaffold protein through its two PDZ domains (1), regulating a broad spectrum of cellular processes such as exosome biogenesis (2), epithelial–mesenchymal Transition (EMT) (3), angiogenesis (4) and various signal transduction pathways (5, 6, 7). It has been shown that syntenin-1 regulates Wnt, EGFR, Integrin, and TGF-β signaling pathways (5, 6, 7). Overexpression of syntenin-1 has been observed in breast cancer, prostate cancer, metastatic melanoma, glioblastoma, head and neck cancers, gastric cancer, liver cancer, urothelial cancer, small cell lung cancer, and esophageal squamous cell cancer (8, 9, 10, 11, 12). Syntenin-1 functions as an oncogene to promote cancer growth and metastasis. The pharmacological inhibitor of syntenin-1, PDZ1i, has been developed to suppress the activity of syntenin-1 through blocking its PDZ domain (13, 14). PDZ1i showed promising anticancer effects in glioblastoma, neuroblastoma, and prostate cancer, used alone or in combination with radiation therapy (15, 16, 17).

Although syntenin-1 emerges as a promising anticancer target and a central hub for various important signaling pathways, its regulatory mechanism is still elusive, especially how its biological activities are regulated by different posttranslational modifications. Several kinases have been reported to phosphorylate syntenin-1, including Src, AURKA, and ULK1. Src-catalyzed syntenin-1 Y4 phosphorylation is involved in immune cell chemotaxis. Src also phosphorylates syntenin-1 at Y46, which promotes exosome biogenesis and cell-to-cell communication (2). AURKA is a Ser/Thr kinase that phosphorylates syntenin-1 at S131 and T200, blocking its ubiquitination and thus stabilizing syntenin-1 protein (18). The other Ser/Thr kinase ULK1 mediates the phosphorylation of syntenin-1 S6 and S61, but the biological function of such phosphorylation events has not been investigated in detail (19).

TANK-binding kinase 1 (TBK1) is a serine/threonine kinase that is essential in regulating innate immunity (20), autophagy (21), and Ras-driven carcinogenesis (22). Dysregulation of TBK1 activity has been associated with the occurrence of multiple types of cancers, including NSCLC (23). It has been demonstrated that TBK1 phosphorylates AGO2 and promotes oncogenic microRNA-induced silencing complex (miRISC) formation, contributing to NSCLC progression. Considering the critical role of TBK1 in NSCLC development, molecular mechanisms, including the specific substrates of TBK1, need to be further addressed.

In this study, while we were pursuing the answer behind syntenin-1 posttranslational modifications, we unexpectedly identified TBK1 as the binding partner of syntenin-1 and then confirmed that TBK1 was the kinase that directly phosphorylated syntenin-1 at S6. We further demonstrated that the TBK1-catalyzed syntenin-1 phosphorylation promoted NSCLC development. Our work validated TBK1 as a new kinase for syntenin-1 phosphorylation and illustrated the role of TBK1-catalyzed syntenin-1 phosphorylation in regulating NSCLC progression.

## Results

### Identification and validation of TBK1 interaction with syntenin-1

**Figure 1:**
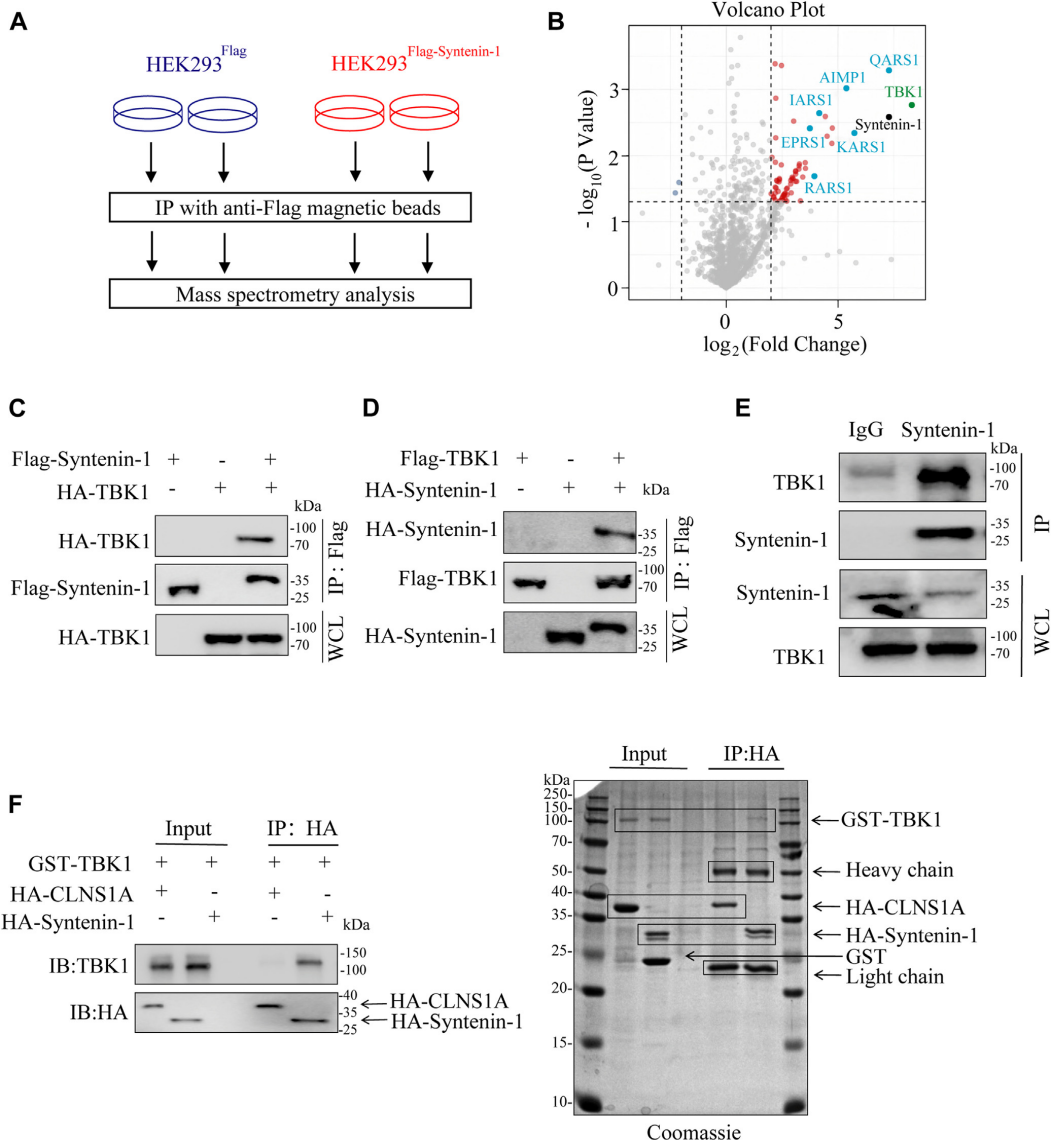
**Identification and validation of TBK1 interaction with syntenin-1.***A*, schematic display of the IP-MS proteomics experiment of syntenin-1 interactome. *B*, volcano plot of syntenin-1 IP-MS proteomics data. The *black dot* represents syntenin-1 bait, the *green dot* represents TBK1, and the *blue dots* represent MARS members. *C*, immunoblots (IBs) of whole cell lysates (WCLs) or immunoprecipitation (IP) products of WCLs of HEK293T cells transfected with plasmids expressing HA-tagged TBK1 and Flag-tagged syntenin-1. *D*, IBs of WCL or IP samples of HEK293T cells transfected with plasmids expressing Flag-tagged syntenin-1 and HA-tagged TBK1. *E*, detection of endogenous syntenin-1 interaction with endogenous TBK1 in A549 cells. *F*, *in vitro* pulldown assay to analyze the interaction between syntenin-1 and TBK1 using purified recombinant GST-TBK1 and HA-syntenin-1, and HA-CLNS1A was utilized as the negative control. The result was analyzed by WB and Coomassie staining. The data shown are a representative result of 3 independent biological replicates.

**Fig. S1:**
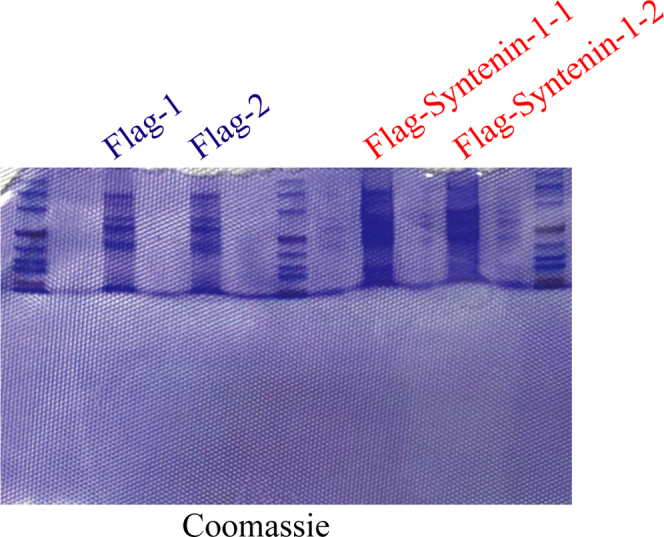
Coomassie staining of IP samples of FLAG alone or FLAG-syntenin-1 used for mass spectrometry analysis.

**Fig. S2:**
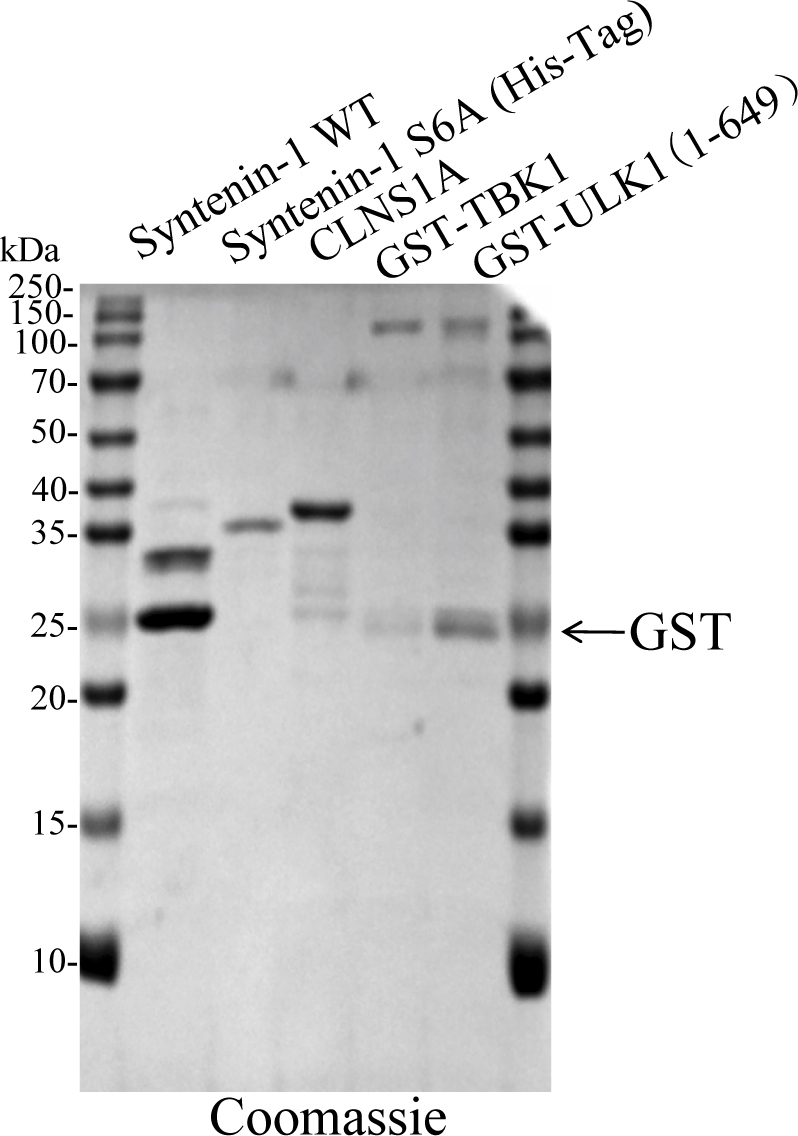
Coomassie staining of all purified recombinant proteins used in this study.

To identify enzymes that catalyze syntenin-1 posttranslational modifications, we performed IP-MS proteomics for Flag-tagged syntenin-1 recombinantly expressed in HEK293T cells (Figs. 1*A*, S1*A*). Using HEK293T cells transfected with empty pcDNA3.4 plasmids as the negative control, our mass spec data identified a bunch of potential syntenin-1 interactors that were significantly enriched in cell expressing Flag-syntenin-1 but not in those expressing Flag tag alone (Fig. 1*B*). Among the identified syntenin-1 interactors, there were known syntenin-1 interacting proteins, including multiple members of the multi-tRNA synthetase complex (MSC) (24, 25, 26), which have been reported to be regulated by syntenin-1 to secrete into the tumor microenvironment to promote inflammation (27). The fact that our IP-MS experiment identified the well-characterized syntenin-1 interacting proteins suggested the reliability of the data. In addition to those MSC proteins, a serine/threonine kinase TBK1, was also among the top hits in our dataset, indicating TBK1 as a syntenin-1 interactor and potential regulator. To validate syntenin-1 interaction with TBK1, Flag-syntenin-1 and HA-TBK1 were recombinantly expressed in HEK293T cells, and coIP experiments showed syntenin-1 was able to pull down TBK1 (Fig. 1*C*), and *vice versa* (Fig. 1*D*). Subsequently, a coIP experiment using an anti-syntenin-1 antibody demonstrated the binding of endogenous syntenin-1 and TBK1 in A549 NSCLC cells (Fig. 1*E*). Additionally, we carried out pull-down experiments using purified GST-HA-syntenin-1 and GST-TBK1 proteins. Purified GST-HA-CLNS1A, an unrelated protein, was used as the negative control (Coomassie staining of purified proteins used in this study is shown in Fig. S2). As shown in Figure 1*F*, GST-HA-syntenin-1 was able to pull down TBK1 while the negative control could not, indicating direct interaction of syntenin-1 and TBK1. To this end, we have identified and validated the binding of TBK1 and syntenin-1.

### TBK1 is a kinase that directly phosphorylates syntenin-1

**Figure 2:**
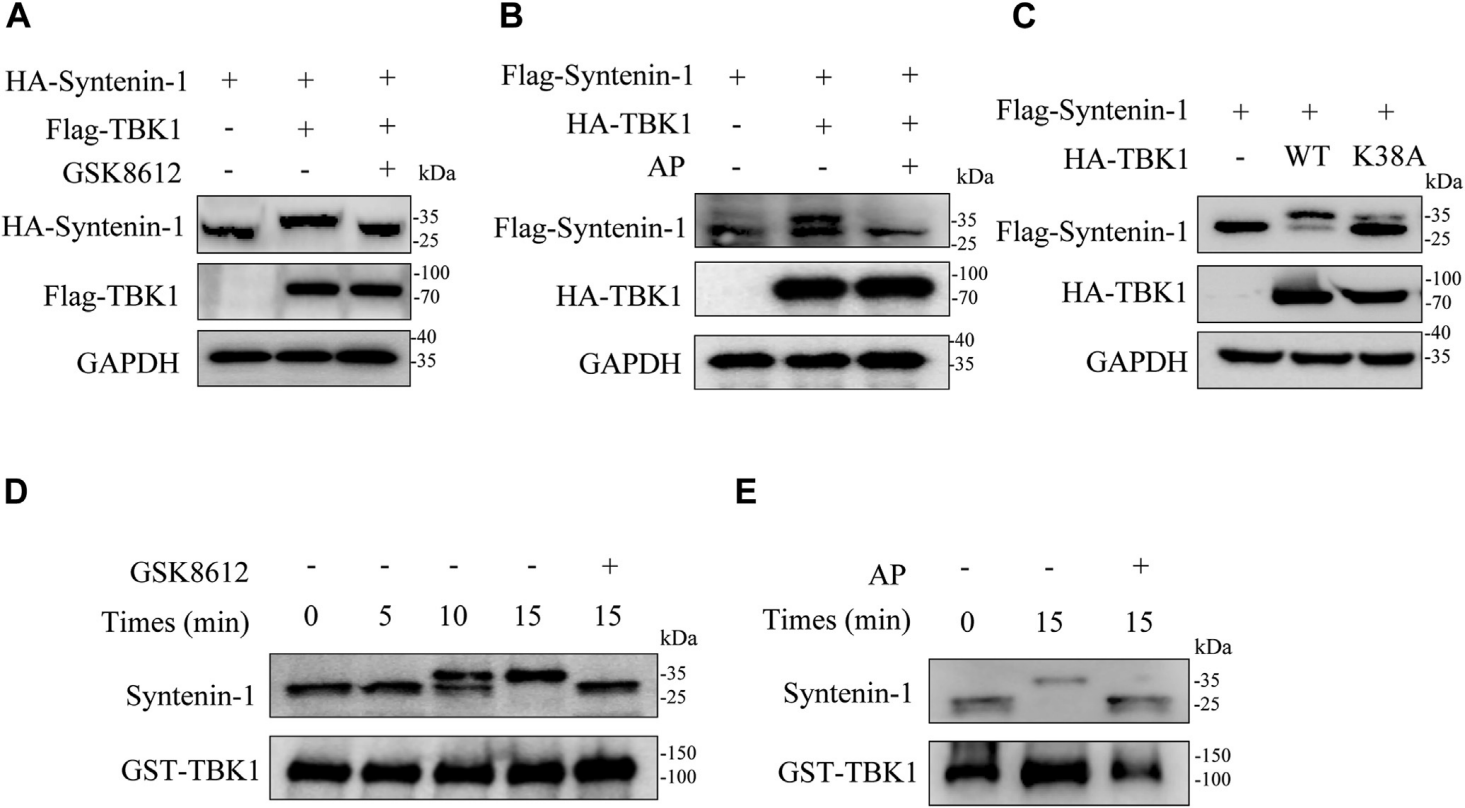
**TBK1 is a kinase that directly phosphorylates syntenin-1.***A*, Western blot analysis of syntenin-1 and TBK1 in HEK293T cells transfected with specific plasmids and treated with DMSO or 1 μM GSK8612. GSK8612: a TBK1 inhibitor. *B*, Western blot analysis of HEK293T cells transfected with Flag-syntenin-1 and HA-TBK1 plasmids and treated with 5 U of alkaline phosphatase (AP) after collection. *C*, IBs of WCL or IP samples of HEK293T cells transfected with plasmids expressing Flag-tagged syntenin-1 and HA-tagged WT or catalytically dead mutant of TBK1. *D*, *in vitro* kinase assay of purified recombinant syntenin-1 and TBK1, with phosphorylation bands of syntenin-1 identified through Western blot analysis, and GSK8612 was added to inhibit the activity of TBK1. *E*, *in vitro* kinase assay of syntenin-1 and TBK1, and AP was added to hydrolyze phosphorylated residues on substrate proteins. The data shown are a representative result of 3 independent biological replicates.

As we have observed syntenin-1-TBK1 interaction, we wondered whether TBK1 was a kinase that could catalyze syntenin-1 phosphorylation. Indeed, when we co-expressed TBK1 with syntenin-1, an apparent upper shift of syntenin-1 band was observed compared to the lane with syntenin-1 alone and this shift could be reversed by adding TBK1 inhibitor GSK8612 (Fig. 2*A*). It is assumable that this band shift is caused by syntenin-1 phosphorylation by TBK1 co-expression, since it is very common that protein phosphorylation leads to slower migration in SDS-PAGE (28). To further confirm that TBK1 co-expression induced syntenin-1 band shift is due to its phosphorylation, we treated the TBK1-syntenin-1 co-expression cell lysate with alkaline phosphatase and the shift of syntenin-1 band disappeared (Fig. 2*B*). Additionally, when syntenin-1 was co-expressed with the catalytically dead mutant of TBK1, the band shift was not observed, suggesting syntenin-1 phosphorylation was dependent on the kinase activity of TBK1 (Fig. 2*C*).

**Fig. S3:**
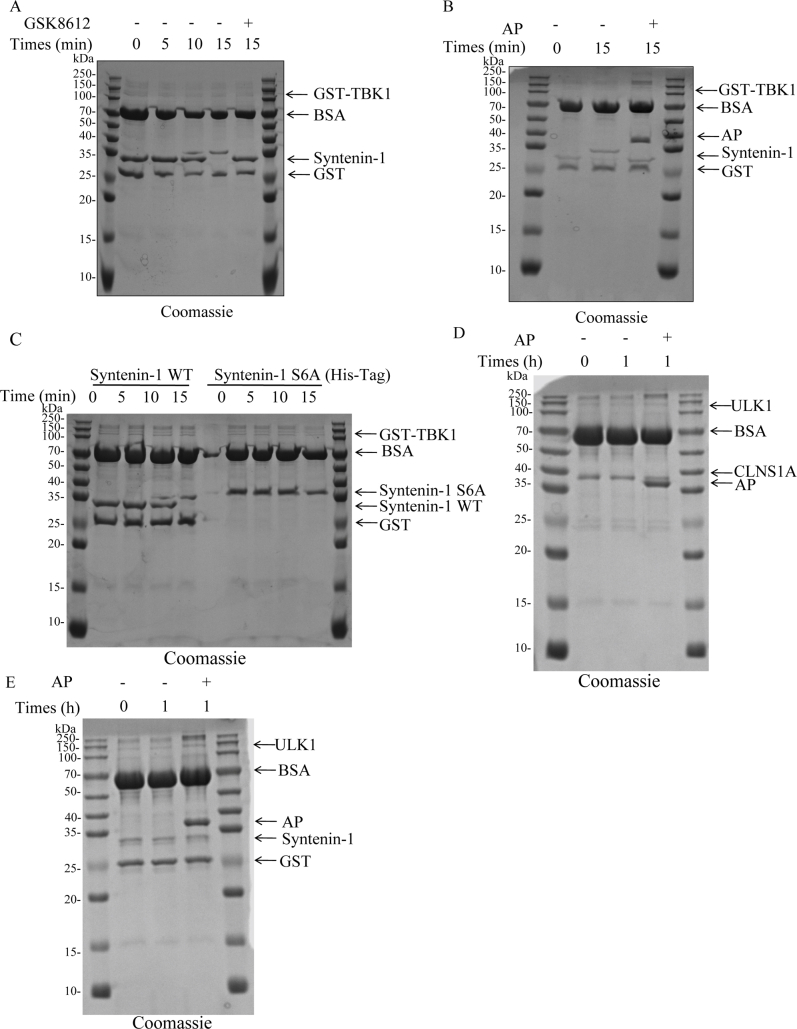
Coomassie staining of the *in vitro* kinases assay samples. (A). *In vitro* kinase assay of TBK1 phosphorylating WT syntenin-1 without or with GSK8612 inhibition. (B). *In vitro* kinase assay of syntenin-1 and TBK1, with AP treatment. (C). *In vitro* kinase assay of syntenin-1 WT/S6A and TBK1. (D). *In vitro* kinase assay of CLNS1A and ULK1. (E). *In vitro* kinase assay of syntenin-1 and ULK1.

In addition to the cell-based experiments, we went on to confirm that TBK1 could directly phosphorylate syntenin-1 with *in vitro* kinase assays using purified recombinant syntenin-1 and TBK1 (Fig. S2). The kinase reaction was performed, and phosphorylation of syntenin-1 was evaluated by the band shift of syntenin-1 in Western blot analysis. As shown in Figures 2*D* and S3*A*, we observed time-dependent phosphorylation of syntenin-1 in the presence of TBK1, and this could be inhibited by GSK8612. Consistently, the band shift of syntenin-1 could be reversed by treatment with alkaline phosphatase, confirming the phosphorylation events (Figs. 2*E* and S3*B*). These results provided robust evidence that TBK1 directly phosphorylated syntenin-1.

### TBK1 phosphorylates syntenin-1 predominantly at S6

**Figure 3:**
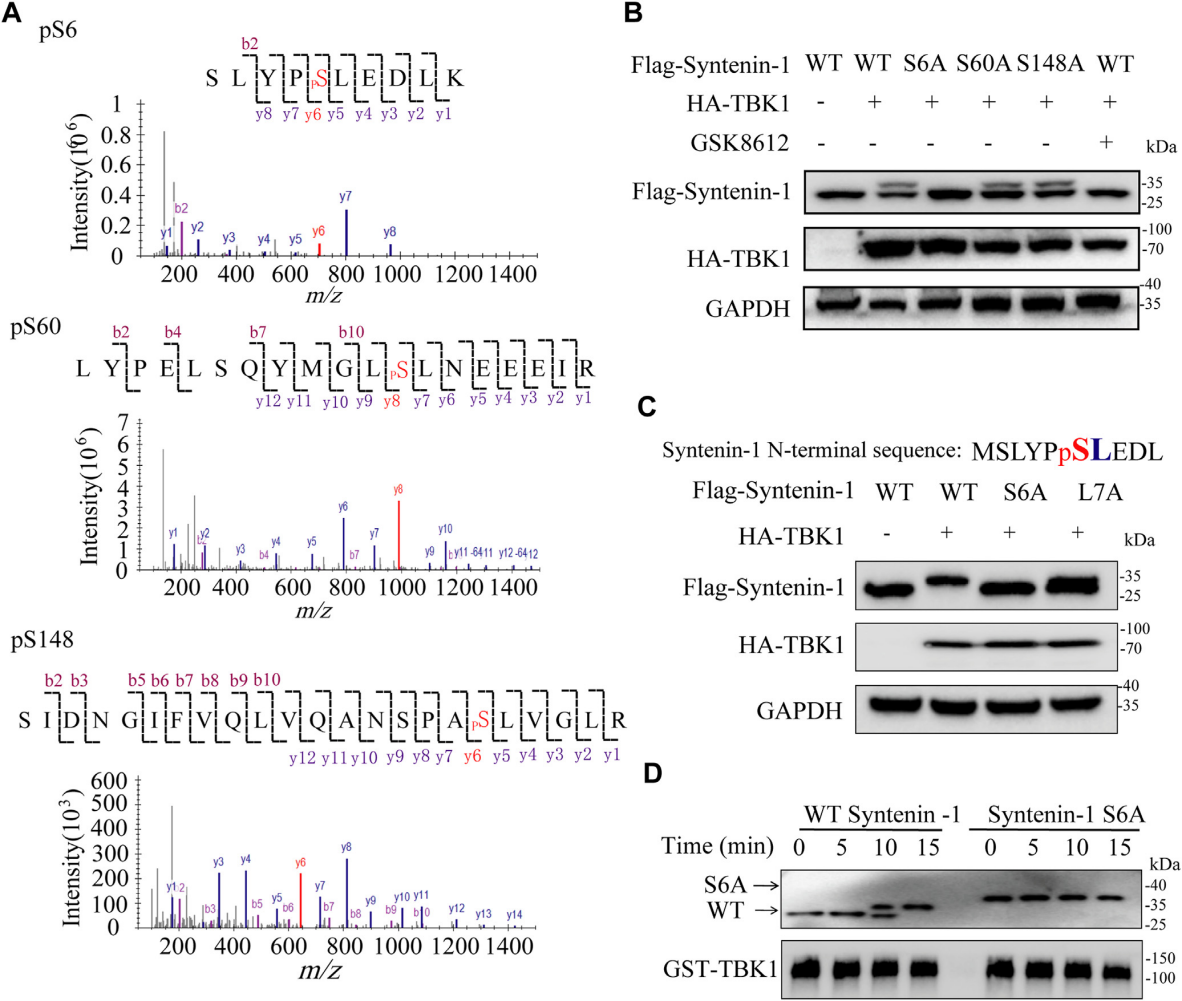
**TBK1 phosphorylates syntenin-1 predominantly at S6.***A*, spectrums of phosphorylated syntenin-1 peptides from samples with syntenin-1-TBK1 co-expression identified by MS analysis. *B*, IBs of WCLs of HEK293T cells transfected with plasmids expressing Flag-tagged TBK1 and HA-tagged syntenin-1 with specific phospho-null mutations. *C*, IBs of WCLs of HEK293T cells transfected with plasmids expressing Flag-tagged WT/S6A/L7A(the residue with large hydrophobic chain required for TBK1 substrate specificity) and HA-tagged TBK1. *D*, *in vitro* kinase assay of WT/S6A syntenin-1 catalyzed by TBK1 detected by WB. The data shown are a representative result of 3 independent biological replicates.

To figure out the syntenin-1 residues phosphorylated by TBK1, we performed mass spectrometry analysis of immunoprecipitated syntenin-1 from cells without or with TBK1 co-expression. S6, S60, and S148 were identified as phosphorylated residues in response to TBK1 co-expression (Fig. 3*A*). We then individually mutated these three residues to alanine and evaluated their phosphorylation status when co-expressed with TBK1. It turned out that S6 was the major phosphorylation site because only the S6A mutation drastically mitigated the phosphorylation by TBK1, while the other two mutants showed little change compared to wildtype (Fig. 3*B*). As TBK1 has been reported to prefer substrates in which a hydrophobic side chain immediately follows the residue being modified (29), we mutated the Leucine residue after S6 in syntenin-1 and observed consistently diminished syntenin-1 phosphorylation of the L7A mutant (Fig. 3*C*). Additionally, *in vitro* kinase assay was carried out with the recombinant S6A-syntenin-1 and showed no phosphorylation by TBK1 (Figs. 3*D* and S3*C*). These results confirmed S6 as the major site of syntenin-1 phosphorylation by TBK1.

### TBK1, rather than ULK1, phosphorylates syntenin-1

Interestingly, it was reported that ULK1 is the kinase that phosphorylates syntenin-1 at residues S6 and S60, overlapping with the sites catalyzed by TBK1 identified here (19, 30). The previous work demonstrated that syntenin-1 could be phosphorylated when ULK1 was co-expressed recombinantly in mammalian cells. However, considering the fact that TBK1 could be phosphorylated and activated by ULK1 (31, 32), it remained an intriguing question whether ULK1 directly catalyzed syntenin-1 phosphorylation or indirectly by phosphorylating and activating TBK1.

**Figure 4:**
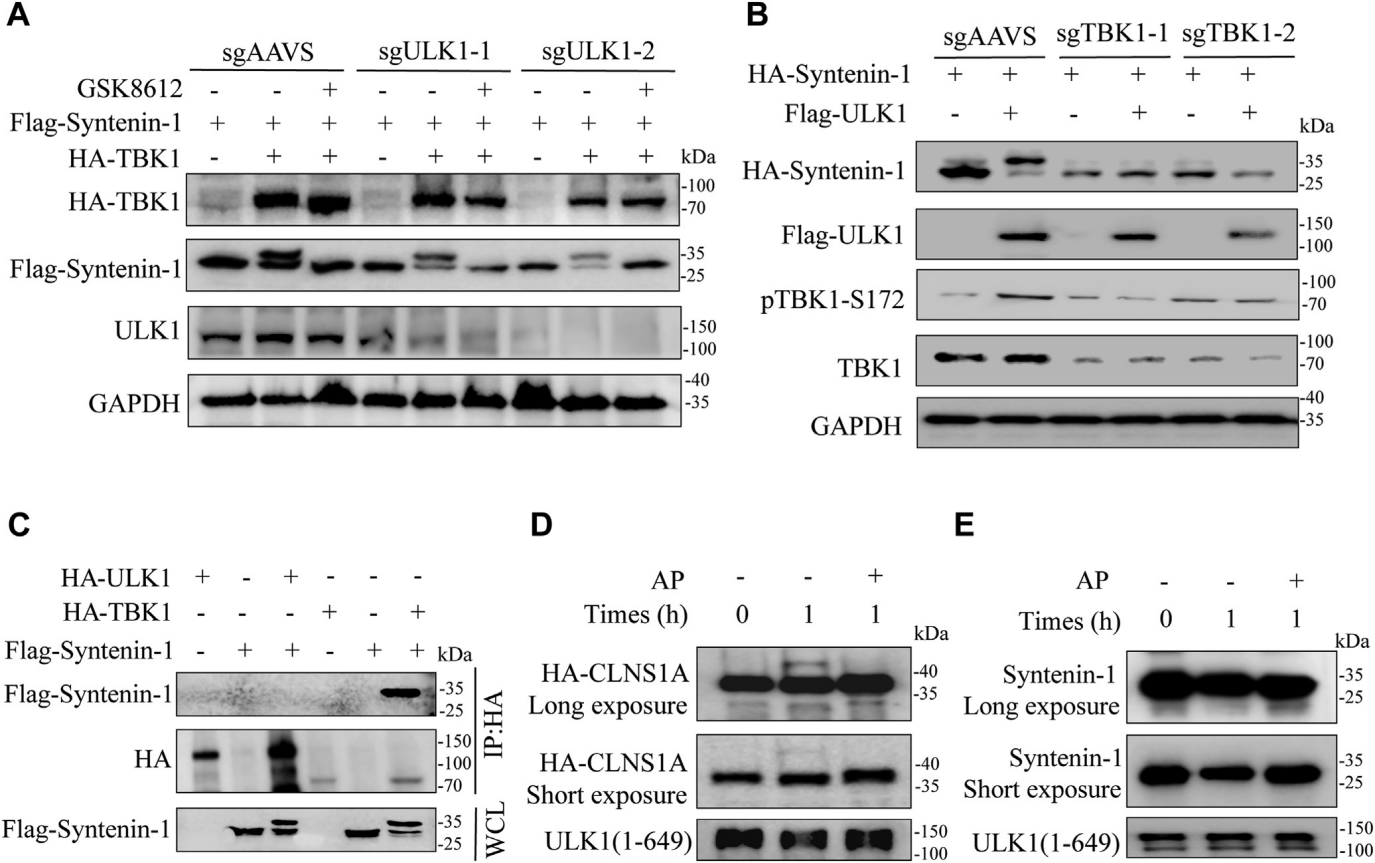
**TBK1, rather than ULK1, phosphorylates syntenin-1.***A*, IBs of WCLs from HEK293T cells infected with lentivirus carrying AAVS or ULK1 sgRNAs, followed by transfection of Flag-syntenin-1 and HA-TBK1. *B*, IBs of WCLs from HEK293T cells infected with lentivirus carrying AAVS or TBK1 sgRNAs, followed by transfection of Flag-tagged ULK1 and HA-tagged syntenin-1. *C*, IBs of WCLs or IP samples of WCLs of HEK293T cells transfected with plasmids expressing Flag-tagged syntenin-1 and HA-tagged-TBK1 or HA-tagged ULK1. *D*, *in vitro* kinase assay of CLNS1A and ULK1, with phosphorylation of CLNS1A indicated by the upward band shift in WB analysis. *E*, *in vitro* kinase assays of syntenin-1 and ULK1. The data shown are a representative result of 3 independent biological replicates.

Based on our findings so far, our IP-MS experiment identified TBK1 as a binding partner of syntenin-1 while ULK1 was not in the list, and our *in vitro* kinase assay provided strong evidence supporting TBK1 directly phosphorylating syntenin-1, we thus speculated that TBK1 is the kinase for syntenin-1 instead of ULK1. To test our hypothesis, we knocked out ULK1 using CRISPR gene editing and analyzed syntenin-1 phosphorylation with TBK1 co-expression. Indeed, syntenin-1 phosphorylation by TBK1 remained the same without the presence of ULK1, indicating ULK1 is not necessary for syntenin-1 phosphorylation (Fig. 4*A*). We also knocked out TBK1 and tested syntenin-1 phosphorylation with ULK1 co-expression. In this case, ULK1 co-expression induced TBK1 and syntenin-1 phosphorylation in wildtype cells but not in TBK1 knockout cells (Fig. 4*B*). IP-WB was performed to investigate the interaction of syntenin-1 with ULK1, and the result showed that syntenin-1 was not able to pull down ULK1 (Fig. 4*C*). Furthermore, we conducted *in vitro* kinase assay of ULK1 to investigate its enzymatic activity towards recombinant syntenin-1. While ULK1 was able to catalyze the phosphorylation of CLNS1A, a known ULK1 substrate (33) (Figs. 4*D* and S3*D*), it could not phosphorylate syntenin-1 (Figs. 4*E* and S3*E*). In sum, our data suggested that it is not ULK1 but rather TBK1 that phosphorylated syntenin-1 at S6.

### Co-expression of TBK1 and syntenin-1 promoted NSCLC progression

**Figure 5:**
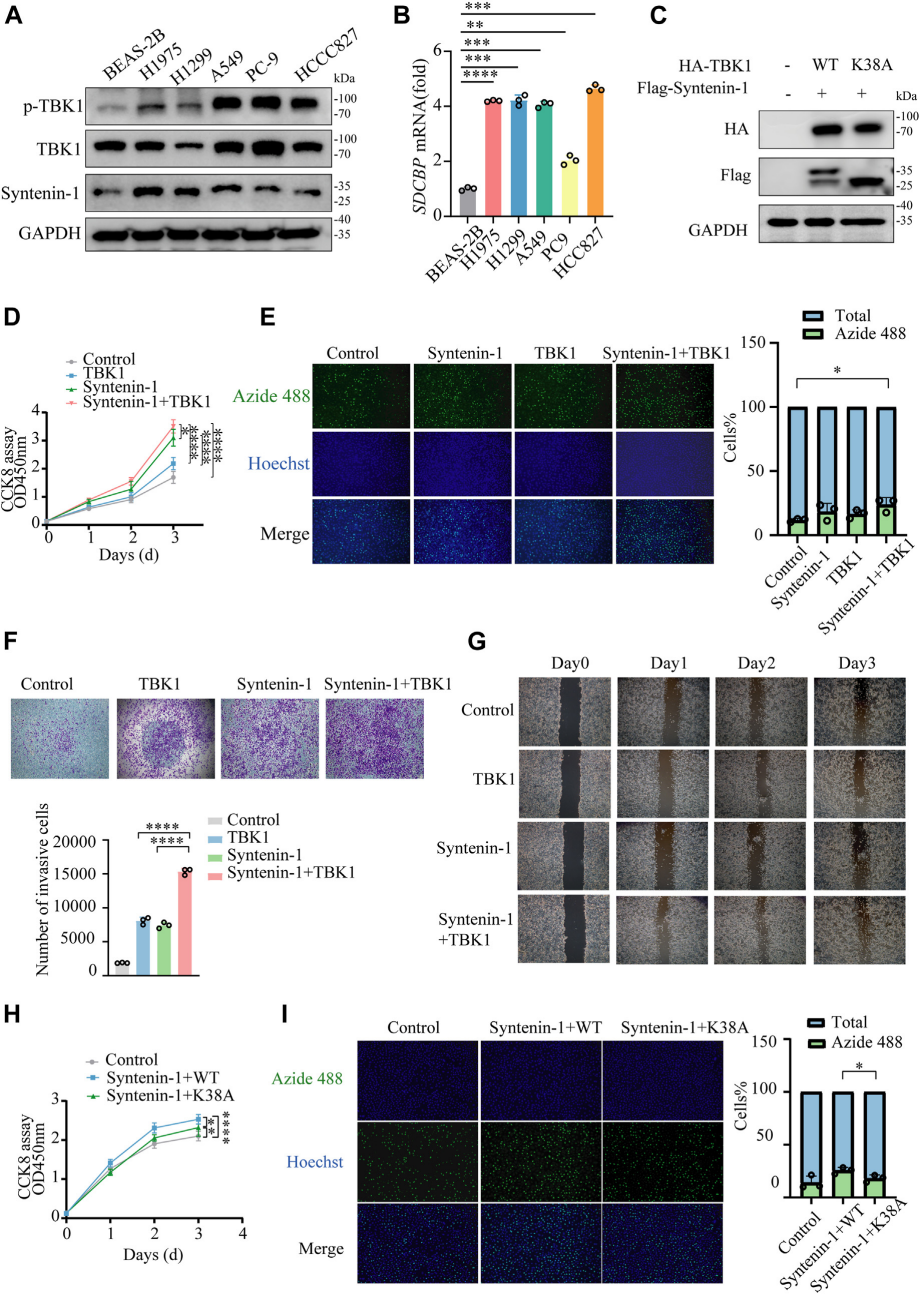
**Co-expression of TBK1 and syntenin-1 promoted NSCLC progression.***A*, IB analysis of syntenin-1, p-TBK1 and TBK1 in BEAS-2B and several NSCLC cell lines. *B*, qPCR analysis of SDCBP mRNA from BEAS-2B and several NSCLC cells. *C*, IB analysis of A549 cells co-expressing Flag-tagged syntenin-1 and HA-tagged WT or catalytically dead K38A mutant TBK1. *D*, CCK-8 assay to measure cell growth of A549 cells with TBK1 and syntenin-1, alone or together. Growth curves of A549 cells were shown for each treatment at 1, 2 and 3 days. *E*, the EdU Cell Proliferation Experiment of A549 cells with single TBK1/syntenin-1 expression or co-expression. *F*, transwell assay of A549 cells transfected with respective plasmids. *G*, wound healing assay to assess the migratory capability of A549 cells transfected with respective plasmids. *H*, CCK-8 assay to measure cell growth of A549 cells transfected with Syntenin-1 and WT or K38A TBK1. Growth curves of A549 cells were shown for each treatment at 1, 2 and 3 days. *I*, the EdU Cell Proliferation Experiment of A549 cells co-transfected with syntenin-1 and WT/K38A TBK1. The two-way ANOVA multiple comparisons test (*D* and *H*) or two-tailed unpaired Student *t* test (*B*, *E*, *F*, *I*) was used for statistical analysis; ∗*p* < 0.05, ∗∗*p* < 0.01, ∗∗∗*p* < 0.001, and ∗∗∗∗*p* < 0.0001. The bars show the mean and SD of n = 3. The data shown are a representative result of 3 independent biological replicates.

We further wondered what biological and pathological functions the TBK1-catalyzed syntenin-1 phosphorylation was. Both syntenin-1 and TBK1 have been reported to be upregulated in multiple cancer types and contribute to cancer progression (34, 35), but none has related those two genes in cancer development. As we have convincingly proved syntenin-1 as a substrate for TBK1 kinase, we continued to investigate the role of syntenin-1 phosphorylation by TBK1 in cancer biology. Using NSCLC as the cancer model, we observed that syntenin-1 protein level was significantly elevated in five NSCLC cell lines compared to the normal lung epithelial cell line BEAS-2B (Fig. 5*A*). It is notable that TBK1 S172 phosphorylation, which is a signature for TBK1 activation (36), was also upregulated in those NSCLC cell lines (Fig. 5*A*). Additionally, qRT-PCR confirmed elevated expression of syntenin-1 at the transcription level in NSCLC cell lines (Fig. 5*B*). These results showed that TBK1 activity and syntenin-1 expression were upregulated in malignant NSCLC cells, indicating a potential role of syntenin-1 and its phosphorylation by TBK1 in NSCLC progression.

**Fig. S4:**
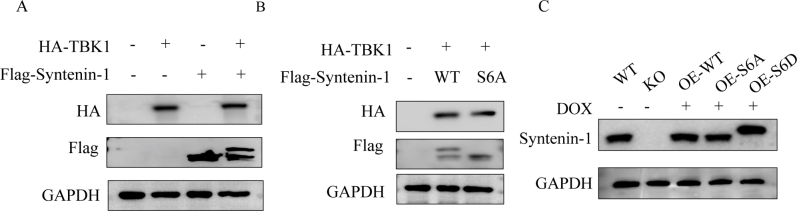
Validation of syntenin-1 and TBK1 overexpression in A549 cells. (A). Western blot analysis of syntenin-1 and TBK1 in A549 cells transfected with specific plasmids. (B). Western blot analysis of WT/S6A syntenin-1 and TBK1 in HEK293T cells transfected with specific plasmids. (C). Western blot analysis of syntenin-1 and TBK1 in syntenin-1-KO A549 cells with WT/S6A/S6D-syntenin-1 re-expression.

We selected A549 as the NSCLC cell line for further investigation as it displayed the most significant upregulation of syntenin-1 expression. Consistent with the results we observed in HEK293T cells, co-expression of syntenin-1 with wildtype TBK1, but not the catalytically dead K38A mutant, led to syntenin-1 phosphorylation (Fig. 5*C*). Cell viability assay using CCK8 showed that co-expression of syntenin-1 and TBK1 could further promote A549 cells growth compared to individually overexpressing syntenin-1 or TBK1 (Figs. 5*D* and S4*A*). To figure out the effect of TBK1-syntenin-1 co-expression on cell proliferation, we employed 5-ethynyl-2′-deoxyuridine (EdU), a thymidine analog, that could be incorporated into newly synthesized DNA during S phase and labeled by click chemistry to add fluorescent tags. The result indicated that A549 cells with TBK1-syntenin-1 co-expression displayed significantly elevated proportion of actively proliferating cells (Fig. 5*E*). Moreover, since syntenin-1 has been reported to play a key role in cancer metastasis, we also tested if TBK1-syntenin-1 co-expression could promote A549 cells migration. As shown by Transwell assay (Fig. 5*F*) and wound-healing assay (Fig. 5*G*), TBK1-syntenin-1 co-expression most significantly induced A549 migration and invasion compared to expressing TBK1 or syntenin-1 alone. To demonstrate that the oncogenic effect of TBK1/syntenin-1 co-expression is dependent on the kinase activity of TBK1, we further evaluated cell growth of A549 cells when syntenin-1 was co-expressed with K38A-TBK1 (Fig. 5*C*). Indeed, the catalytically inactive TBK1 was much less potent than wildtype to promote cell growth and proliferation (Fig. 5, *H* and *I*). These results clearly demonstrated that TBK1-syntenin-1 co-expression could promote A549 cancer cell growth and migration, indicating the role of TBK1 mediated syntenin-1 phosphorylation in NSCLC progression.

### S6 phosphorylation by TBK1 sustains syntenin-1 oncogenic function in NSCLC

**Figure 6:**
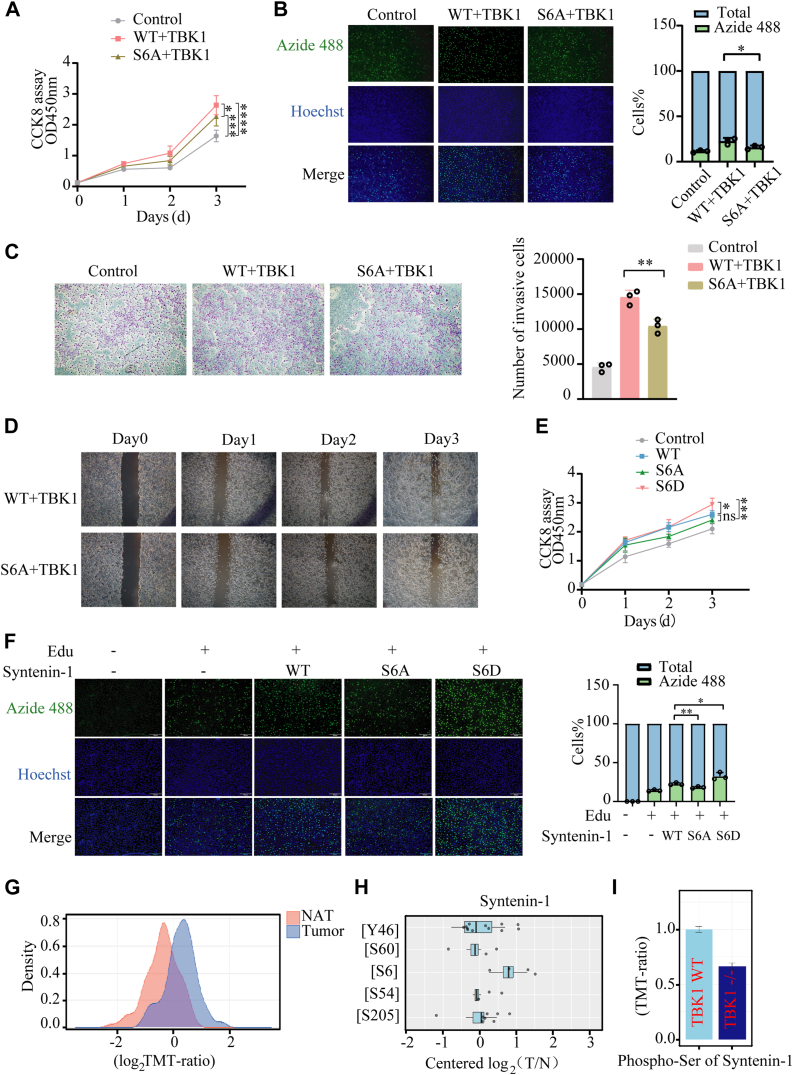
**S6 phosphorylation by TBK1 sustains syntenin-1 oncogenic function in NSCLC.***A*, CCK-8 assay to measure cell growth of A549 cells transfected with WT/S6A syntenin-1 and TBK1. Growth curves of A549 cells were shown for each treatment at 1, 2 and 3 days. *B*, the EdU Cell Proliferation Experiment of A549 cells with TBK1 and WT/S6A-syntenin-1 co-expression. *C*, transwell assay for assessing the invasive capacity of A549 cells transfected with respective plasmids. *D*, scratch assay to assess the migratory capability of A549 cells transfected with respective plasmids. *E*, CCK-8 experiment to assess cell growth of syntenin-1 knockout A549 cells that recombinantly expressed WT/S6A/S6D-syntenin-1. Growth curves of A549 cells were shown for each condition at 1, 2, and 3 days. *F*, EdU analysis of cell proliferation of the A549 cells under the conditions in (*E*). *G*, density plot of phosphorylated syntenin-1/nonphosphorylated syntenin-1 ratios in NSCLC tumor samples and normal adjacent tissue (NAT) control samples, derived from data of TMT quantitative proteomics studies of human clinical samples. *H*, relative quantification of syntenin-1 phosphorylation sites in NSCLC tumor samples compared to NAT controls. *I*, analysis of the phosphorylation levels of syntenin-1 in the WT TBK1 and TBK1−/− groups. The two-way ANOVA multiple comparisons test (*A* and *E*) or two-tailed unpaired Student *t* test (*B*, *C*, *F*) was used for statistical analysis; ∗*p* < 0.05, ∗∗*p* < 0.01, ∗∗∗*p* < 0.001, and ∗∗∗∗*p* < 0.0001. The bars show the mean and SD of n = 3. The data shown are a representative result of 3 independent biological replicates.

Knowing that TBK1-syntenin-1 co-expression could drive NSCLC progression, we want to confirm that this is due to the phosphorylation events of syntenin-1 by TBK1. To address this, we evaluated A549 cells growth and migration when TBK1 was co-expressed with wild-type or S6A mutant syntenin-1 (Fig. S4*B*). The CCK8 and EdU assay results showed that the phosphor-null S6A-syntenin-1 was less potent than wildtype to promote A549 cells' growth and proliferation (Fig. 6, *A* and *B*). Transwell assay and wound healing assay revealed a similar trend that A549 cells with TBK1 and wildtype syntenin-1 co-expression displayed more significant migration than the phospho-null mutant (Fig. 6, *C* and *D*). To further address this question with a cleaner background, we utilized CRISPR gene editing to knock out syntenin-1 in A549 cells and then recombinantly express WT, S6A, and S6D-syntenin-1 (Fig. S4*C*). It was shown that S6D-syntenin-1 most significantly promoted cell growth and proliferation (Fig. 6, *E* and *F*). These results showed that TBK1 catalyzed syntenin-1 S6 phosphorylation could potentiate the oncogenic activity of syntenin-1 in A549 cells.

To investigate syntenin-1 phosphorylation state in the samples of NSCLC patients, bioinformatic analysis was performed with the phosphoproteomics datasets of more than one hundred NSCLC tumor samples and matched to normal adjacent tissues (NATs) from the CPTAC data portal. We found that the phosphorylation levels of syntenin-1 serine residues in NSCLC tumors were significantly elevated compared to the NAT controls (Fig. 6*G*) (37). Moreover, phosphorylation of S6 was the highest among those syntenin-1 phosphor-serine sites in NSCLC tumors (Fig. 6*H*) (38). Additionally, we utilized the quantitative phosphoproteome data of TBK1 knockout cancer cells to explore the effect of TBK1 on syntenin-1 phosphorylation and observed that the phosphorylation level of syntenin-1 serines was notably downregulated in TBK1 knockout cells compared to wild-type cells (Fig. 6*I*) (39). Such bioinformatic analysis of quantitative phosphoproteomics data from NSCLC clinical samples and cell lines also supported the oncogenic effect of syntenin-1 phosphorylation by TBK1.

### Syntenin-1 S6 phosphorylation activates MAPK signaling in NSCLC cells

**Figure 7:**
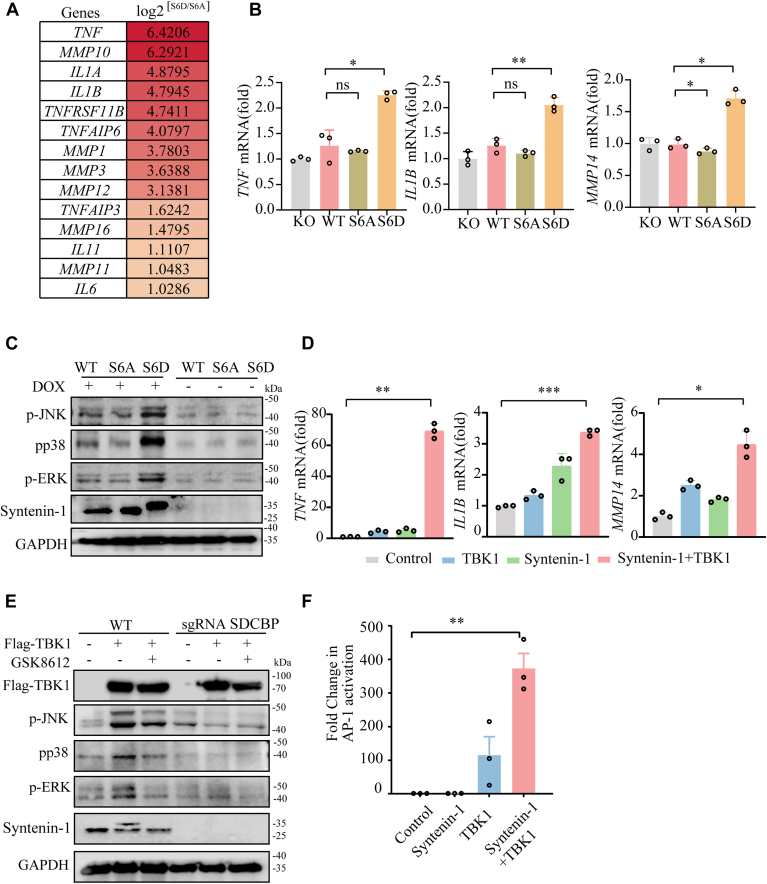
**Syntenin-1 S6 Phosphorylation activates MAPK signaling in NSCLC cells.***A*, heatmap of RNA sequencing data of MAPK downstream target genes in SDCBP -KO A549 cells re-expressing S6A or S6D syntenin-1. *B*, qPCR analysis of *TNF*, *IL1B* and *MMP14* mRNA from SDCBP knockout A549 cells that recombinantly expressed WT/S6A/S6D-syntenin-1. *C*, after 2 days of DOX induction, phosphorylation of JNK, ERK, and p38 was detected by Western blot. *D*, qPCR analysis of *TNF*, *IL1B,* and *MMP14* mRNA from A549 cells transfected with respective plasmids. *E*, Western blot detection of the phosphorylation of JNK, ERK, and p38 in WT and SDCBP knockout A549 cells transfected with TBK1. *F*, luciferase assay of AP-1 reporter gene in A549 cells overexpressing TBK1 and syntenin-1. The two-tailed unpaired Student *t* test (*B*, *D*, *F*) was used for statistical analysis; ∗*p* < 0.05, ∗∗*p* < 0.01, ∗∗∗*p* < 0.001, and ∗∗∗∗*p* < 0.0001. The bars show the mean and SD of n = 3. The data shown are a representative result of 3 independent biological replicates.

**Fig. S5:**
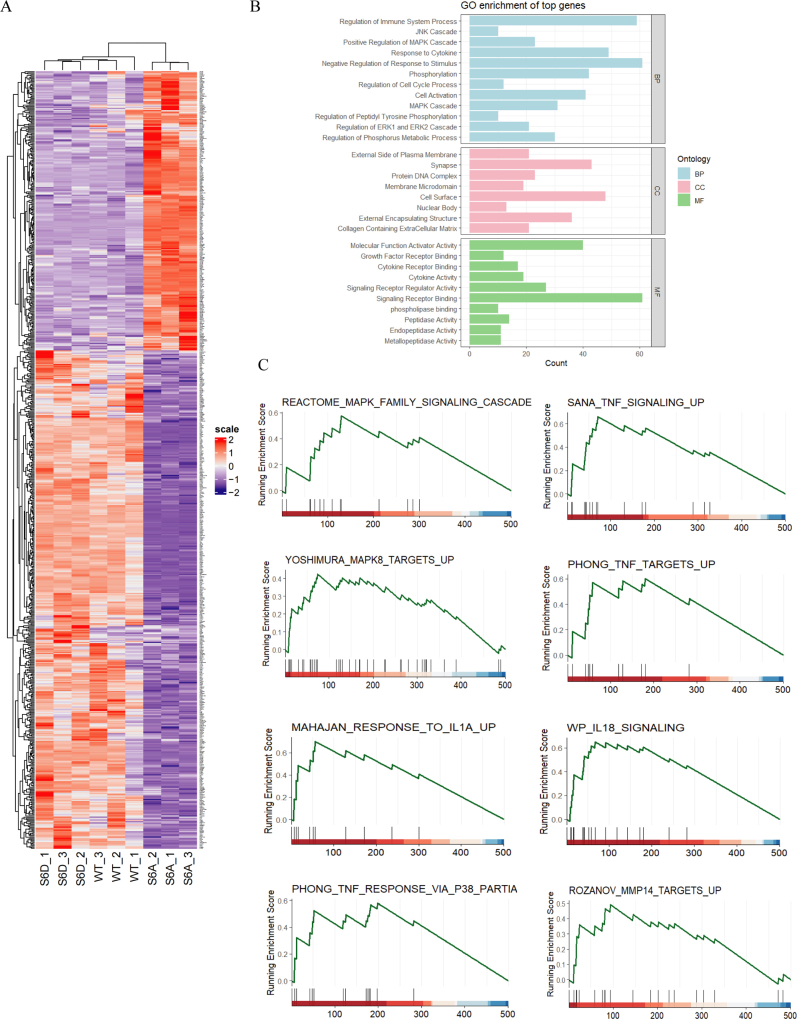
RNA-seq data of syntenin-1-KO A549 cells with WT/S6A/S6D-syntenin-1 re-expression. (A). Heatmap of RNA-seq data presenting the genes with significant changes. (B). GO analysis of top changers in RNA-seq data. (C). GSEA plots of MAPK related pathways.

To explore the overall impact of TBK1-mediated syntenin-1 phosphorylation on cellular signaling and gene expression, RNA sequencing was conducted to evaluate transcriptome changes caused by syntenin-1 S6 phosphorylation. We used syntenin-1 knockout A549 cells that recombinantly express wildtype, S6A and S6D syntenin-1. Analysis of the corresponding transcriptomics data suggested that the MAPK signaling was significantly upregulated by syntenin-1 S6 phosphorylation as indicated by multiple MAPK downstream cytokines with oncogenic implications (Figs. 7*A* and S5). We further validated the RNA sequencing data by quantitative RT-PCR. Consistently, the mRNA levels of *TNF, IL1B* and *MMP14* were significantly elevated in S6D-syntenin-1 group and decreased in S6A-syntenin-1 group compared to WT (Fig. 7*B*). Additionally, Western blotting analysis of MAPK signaling pathway revealed that S6D-syntenin-1 induced more dominant phosphorylation of JNK, p38 and ERK, the key signaling molecules in MAPK pathway, indicating a stronger activation of the MAPK signaling pathway by S6 phosphorylated syntenin-1 (Fig. 7*C*). The effect of TBK1 co-expression on MAPK signaling was also evaluated. MAPK downstream targets including *TNF, IL1B* and *MMP14* were dramatically induced at the transcriptional level by syntenin-1 and TBK1 co-expression (Fig. 7*D*). Moreover, the Western blot analysis revealed that TBK1-syntenin-1 co-expression enhanced phosphorylation of JNK, p38, and ERK (Fig. 7*E*). Luciferase assay demonstrated that TBK1-syntenin-1 co-expression induced the most robust AP-1 activation (Fig. 7*F*). In total, it was indicated by our results that syntenin-1 S6 phosphorylation activated MAPK pathway in A549 cells, which might be the mechanism it promoted NSCLC cell growth and migration.

## Discussion

In this study, we explored the phosphorylation of syntenin-1 by TBK1 and the potential pathological function to potentiate NSCLC progression. Phosphorylation is a well-known post-translational modification that plays a critical role in regulating the activity and function of numerous proteins (18, 40). As syntenin-1 is a promising target for cancer treatment, delineating the kinases that directly phosphorylate syntenin-1 and regulate its function could offer novel therapeutic strategies (41, 42, 43). Our proteomic and biochemical analysis identified and validated TBK1 as the kinase for syntenin-1 S6 phosphorylation. Intriguingly, syntenin-1 S6 phosphorylation has been previously reported to be catalyzed by ULK1 (19), giving rise to an interesting biochemical question: Which one is actually the kinase that directly phosphorylates syntenin-1? A cohort of evidence supports TBK1 as the direct kinase for syntenin-1 instead of ULK1: IP-MS and IP-WB revealed the binding between syntenin-1 and TBK1, but not ULK1; knocking out ULK1 does not affect syntenin-1 phosphorylation; syntenin-1 could be phosphorylated by TBK1 *in vitro,* while ULK1 could not. Considering that ULK1 could phosphorylate and activate TBK1, we propose a ULK1/TBK1/syntenin-1 pathway for the regulation of syntenin-1 phosphorylation. This could explain the results from previous reports that overexpressing ULK1 could lead to syntenin-1 phosphorylation (19), which was probably achieved indirectly by activating TBK1. Our experiments uncovered a more accurate molecular mechanism for the phosphorylation of syntenin-1 S6 phosphorylation and underscore the importance of *in vitro* enzymatic characterization to conclude about protein kinase-substrate relationships.

In addition to characterization of the molecular details of syntenin-1 phosphorylation, our study has revealed syntenin-1 as a new substrate for TBK1. This discovery holds particular significance given TBK1’s emerging role as a therapeutic target in oncology, especially for overcoming resistance to cancer immunotherapy (44). However, the precise molecular mechanisms underlying TBK1’s function in cancer biology, particularly how TBK1 regulates cancer immunology, need to be further investigated. Our findings establish an intriguing connection between TBK1 and exosome regulation through syntenin-1, a key player in exosome biogenesis (45). This may provide mechanistic insights into TBK1’s ability to modulate the tumor microenvironment and cancer immunology. We speculate that TBK1-catalyzed phosphorylation of syntenin-1 could alter its functional capacity in exosome secretion, thereby modulating intercellular communication among tumor cells and immune cells within the tumor microenvironment. Further studies should be carried out to investigate the cellular functions of syntenin-1 phosphorylation by TBK1, particularly its impact on exosome-mediated cellular crosstalk and subsequent immunological effects.

Furthermore, our findings suggest that the phosphorylation of syntenin-1 may have significant implications for cancer progression, particularly in non-small cell lung cancer (NSCLC) (46). syntenin-1, a protein involved in cell adhesion and cytoskeletal organization, could modulate the invasiveness and metastatic potential of cancer cells through phosphorylation events (47, 48). In the case of NSCLC, alterations in syntenin-1 phosphorylation may affect tumor cell proliferation and metastasis. The activation of TBK1 in NSCLC may exacerbate these processes, potentially linking syntenin-1 phosphorylation to the progression of this cancer type. Therefore, investigating the role of syntenin-1 phosphorylation in NSCLC could uncover novel biomarkers or therapeutic targets for improving patient outcomes. Future studies should focus on understanding how specific phosphorylation sites on syntenin-1 contribute to NSCLC progression and whether targeting these modifications can provide clinical benefits in the treatment of this aggressive cancer.

## Experimental procedures

### Cells and antibodies

HEK293T cells (CRL-3216), A549 cells (CCL-185) and BEAS-2B cells (CRL-9609), obtained from American Type Culture Collection (ATCC) were maintained in Dulbecco modified Eagle medium (Gibco, C11995500BT) supplemented with 10% (v/v) fetal bovine serum (Biosera; 010423-UY), penicillin (100 U/ml), and streptomycin (100 μg/ml) solution (Gibco, 15140-122). H1975 cells (CRL-5908), H1299 cells (CRL-5803), HCC827 cells (CRL-2868) and PC-9 cells (CRL-AD0240) obtained from American Type Culture Collection (ATCC) were maintained in RPMI 1640 (Gibco, C11875500BT) supplemented with 10% (v/v) fetal bovine serum (Biosera; 010423-UY), and penicillin (100 U/ml) and streptomycin (100 μg/ml) solution (Gibco, 15140-122). The following antibodies were used for Western blot analysis: anti-phospho-ERK (CST, 9101), anti-phospho-38 (CST, 4511), anti-phospho-SAPK/JNK (CST, 9251), anti-HA horseradish peroxidase (HRP) conjugate (ABclonal, AE025), anti-Flag HRP conjugate (CST, 86861), anti-TBK1 (Proteintech, 28397-1-AP), anti-syntenin-1 (Proteintech, 22399-1-AP), anti-ULK1 (ABclonal, A8529), and anti-phospho-TBK1/NAK(Ser-172) (CST, 5483) were all diluted at a ratio of 1:1000; and anti-GAPDH (ABclonal, AC002) was diluted at a ratio of 1:10,000. For immunoprecipitation experiments, we used BeyoMag Anti-HA Magnetic Beads (Beyotime, P2121) and BeyoMag Anti-Flag Magnetic Beads (Beyotime, P2115). HEK293T cells were transiently transfected using polyethylenimine (Polysciences, 23966-2) for 36 to 48 h, followed by co-immunoprecipitation and Western blot assay. A549 cells were transfected with lipo8000 (Beyotime, C0533), and Western blot analysis or other experiments were conducted 36 to 48 h post transfection. Equal amounts of DNA plasmids were transfected into each well of cells, and empty vector was used when necessary.

### Mass spectrometry sample preparation and analysis

HEK293T cells were transfected with the corresponding plasmids using PEI. After 48 h, cells were harvested by adding proper amount of cell IP lysis solution (which contains protease inhibitors) and incubated for 30 min at 4 °C on ice. After 30 min of centrifugation at 12,000 g, the supernatant was collected. After a small amount of lysis buffer was aliquoted for Western blot analysis, 15 μl of Flag magnetic beads was added to the remaining lysate and incubated overnight with gentle shaking at 4 °C. Subsequently, the mixture was washed three times with PBS-Tris buffer (1% Triton X-100 in PBS) and boiled with 1× sodium dodecyl sulfate (SDS) loading buffer for 10 min. The samples were subject to SDS-PAGE and Coomassie staining before mass spectrometry sample preparation. The gel pieces with protein staining were excised into small pieces and transferred to Eppendorf tubes for further MS sample preparation. The gel pieces were treated with acetonitrile to remove stains and dried with vacuum centrifugation. Subsequently, the gel was treated with 20 mM TCEP (dissolved in 100 mM ammonium bicarbonate) and 50 mM IAA (dissolved in 100 mM ammonium bicarbonate) for reduction and alkylation, respectively. Next, trypsin solution (10 ng/μl) was added to the gel pieces to digest overnight at 37 °C. Following digestion, the peptides were extracted with a series of extraction buffers as following: 5% formic acid, 50% acetonitrile, 70% acetonitrile, and 100% acetonitrile. The samples were then concentrated and dried in vacuum. Salt removal can be performed using C18 Spin Tips to obtain peptide segments, with the peptide concentration adjusted to 0.2 μg/μl. The samples were processed with the Vanquish nanoliter liquid chromatography system with FAIMS Pro and the Orbitrap Exploris 480 mass spectrometer (Thermo Scientific) interfaced for data-dependent Acquisition (DDA). The Swissprot Human database was employed for data analysis.

### Immunoprecipitation and Western blotting

To perform immunoprecipitation, PEI was used to transiently transfect HEK293T cells. After 48 h, the cells were washed with phosphate-buffered saline (PBS) and then lysed in cell lysis buffer for western blotting and immunoprecipitation (Beyotime, P0013) supplemented with 1 mM phenylmethylsulfonyl fluoride (PMSF), 1 mM Na_3_VO_4_, and 1 mM NaF, for 30 min on ice. The lysates were centrifuged at 17,500 g for 10 min at 4 °C to remove the debris. The cell lysates were incubated overnight with anti-Flag magnetic beads at 4 °C. The magnetic beads were isolated with a magnetic rack, washed three times with PBS-Tris buffer (1% Triton X-100 in PBS) and boiled with SDS loading buffer for 10 min. Equivalent amounts of protein were separated using SDS-PAGE and transferred to polyvinylidene difluoride membranes (Bio-Rad, 1620177). The membranes were incubated overnight with the primary antibodies at 4 °C, washed three times with TBST (1% Tween-20; Sangon Biotech, A600560), and then incubated with secondary HRP-conjugated antibodies for 2 h at room temperature. After a final wash with TBST, the proteins were visualized using enhanced chemiluminescence reagent (ABclonal, RM00021).

### Protein purification

The pET28A His-syntenin-1 construct was introduced into BL21 (DE3) E.coli cells and cultured at 37 °C until reaching an optical density at 600 nm (OD600) of 0.8, followed by induction with 0.5 mM IPTG at 18 °C for 18 to 22 h. The cells were then harvested, resuspended in a buffer containing 20 mM Tris pH8.0, 200 mM NaCl, 1 mM PMSF, 1 mM DTT, and additional Protease Inhibitor Cocktail (EDTA-Free). After homogenization and centrifugation, the supernatant was applied to a Ni-NTA bead column, followed by extensive washing with a buffer consisting of 20 mM Tris pH 8.0, 200 mM NaCl. The His tagged protein was eluted with a buffer containing 20 mM Tris pH 8.0, 200 mM NaCl and 500 mM imidazole. The eluted fraction was concentrated to 0.5 ml and purified by size exclusion chromatography (Superdex 200 increase 10/300 Gl) using a buffer of 20 mM Tris pH 8.0, 200 mM NaCl. The purified Human His-syntenin-1 fractions were concentrated with the addition of 5% (v/v) glycerol and stored at −80 °C. The S6A mutant and CLNS1A were expressed and purified following a protocol similar to the one described above.

### *In vitro* kinase assay

*In vitro* kinase assays were performed by incubating 0.75 μg active TBK1 (SignalChem, T02-10G-10) or ULK1 (SignalChem, U01-11G) with 1 μg syntenin-1, syntenin-1 S6A or CLNS1A purified recombinant proteins in kinase assay buffer (20 mM Tris-HCl, pH 7.4, 0.5mg/ml BSA, 10 mM MgCl_2_, 10% glycerol and 0.25 mM dithiothreitol (DTT)) plus 100 μM ATP at 30 °C. ATP was added to initiate the reaction and aliquots of the reaction were quenched by mixing with SDS loading buffer at different time points. After boiling, Western blot analysis was used to evaluate substrate phosphorylation.

### *In vitro* pull-down assay

Purified HA-Sytenin-1 (0.5 μg) or HA-CLNS1A (0.5 μg) was incubated with anti-HA magnetic beads in the buffer containing 20 mM Tris pH 7.5, 150 mM NaCl and 10% glycerol for 1 h at room temperature. Subsequently, 0.25 μg GST-TBK1 protein (SinoBiological, T02-10G) was added to the mixtures and incubated with rotation at 4 °C overnight. After incubation, the magnetic beads were separated using a magnetic rack, and the liquid above the beads was removed. The beads were then washed three times with PBST buffer (1% Triton X-100 in PBS) and boiled for 10 min in SDS loading buffer. The samples were analyzed by both Coomassie staining and Western blot analysis using anti-TBK1 antibody (ABclonal) or anti-HA antibody (ABclonal).

### CRISPR-based gene editing to knock out TBK1, ULK1, and syntenin-1

We utilized Benchling (https://help.benchling.com/hc/en-us) to design sgRNAs. Selected sgRNA sequences were ligated into the lentiCRISPR v2 vector for lentivirus production. The corresponding lentivirus was generated in HEK293T cells transfected with lentiCRISPR v2, psPAX2, and pMD2.G at the ratio of 3:2:1. A549 and HEK293T cells were infected with the lentivirus expressing Cas9 nuclease and sgRNAs with the presence of 0.8 μg/ml polybrene. 1 μg/ml puromycin was then added to select for successfully infected cells. Western blot was used to evaluate the efficiency of knockout for TBK1 and ULK1. For syntenin-1, the infected cells were sorted as single cells into 96-well plates and expanded as single clones. The knockout cells were screened and picked for subsequent experiments. The sgRNA sequences used in this work include the following: sgTBK1-1 (CATAAGCTTCCTTCGTCCAG), sgTBK1-2 (GACAGCAGATTATCTCCAGG); sgULK1-1 (CCACGGTCTCTGTGCCGCCG); sgULK1-2 (CGCGGCGGCACAGAGACCGT); sgSDCBP (ACCTTCAAGTCTTCGAGAGA). sgAAVS (GGGACTCTTTAAGGAAAGA) was used as the negative control.

### The piggybac overexpression system

A piggybac recombination overexpression system was used to recombinantly express syntenin-1 and its mutants in syntenin-1 knockout A549 cells. Specifically, the SDCBP knockout A549 cells were transfected with PB-TE-WT syntenin-1, S6A and S6D plasmids along with the Hypbase plasmids to generate stable cell lines with doxycycline-inducible syntenin-1 expression. Hygromycin was used to select for cells that successfully incorporated the syntenin-1 expressing cassette.

### CCK8 cell growth assay

Cell growth was evaluated using the Cell Counting Kit-8 (Beyotime, C0041). A549 cells were plated in 96-well plates at a density of 1 × 10^4^ cells per well in 100 μl of growth medium and allowed to attach overnight. At various time points (12, 24, 48, and 72 h), the culture medium was aspirated and replaced with 100 μl DMEM culture medium containing 10 μl of CCK-8 solution. After incubation at 37 °C for 1 h, the absorbance values were measured at 450 nm using an ELX-800 spectrophotometer. This assay was performed in triplicate, with six replicates per condition each time.

### Transwell assay

The migratory ability of A549 cells was assessed using Transwell chambers (Corning, 3422). Add 1 × 10^5^ cells transfected with plasmids into each well using 200 μl serum-free medium. 500μl of medium containing 20% fetal bovine serum was added to the lower chamber. After 24 h, the upper surface of the membrane was gently wiped with a cotton swab, and the cells that had migrated to the lower chamber were fixed with 4% paraformaldehyde for 20 min, stained with 0.1% Crystal violet, and then washed with PBS. Cell invasion was observed under a microscope, and cell numbers were quantified using Image J.

### EdU cell proliferation assay

The proliferation of cells was detected using EdU cell proliferation assay according to the manufacturer’s instructions. Firstly, place 12 mm slides in a 12-well plate and seed 3 × 10^5^ cells for cultivation for 24 h. After induction with DOX for 2 days, a total of 500 μl EdU (10 μM) reagent (Beyotime, C0071S) was added to each well and incubated for 2 h to label the cells. After being washed with PBS three times, the cells were fixed in 500 uL Immunostaining fixative (Beyotime, P0098) for 15 min, permeabilized with 0.3% Triton X-100 (GenStar, VA11410) for another 10 min, and incubated with the Adize-488 reaction reagent for 30 min at room temperature in a dark environment. In total, 1 × Hoechst 33342 reagent was utilized for nuclear counterstaining. The staining result was visualized using a fluorescence microscope (OLYMPUS), and the data were acquired through the Image J software.

### Wound-healing assay

A549 cells were plated into a 6-well plate and transfected with plasmids in accordance with the grouping. When the cells reached full confluence, culture medium with low serum (1%) was applied to minimize cell proliferation. Three vertical scratches were introduced using a 200 μl pipette tip and images of the scratches were recorded at different time points after scratching.

### Quantitative real-time RT-PCR RNA

Cellular total RNA was extracted using RNAiso Plus (Takara, 9109) following the manufacturer's protocol. The total RNA was then reverse-transcribed into cDNA using the Primescript RT kit (ABclonal, RK20428). An SYBR RT-PCR kit (ABclonal, RK21203) was used for quantitative real-time polymerase chain reaction (qRT-PCR) analysis. Gene amplification was carried out using the 2 (-ΔΔCt) method. The primer sequences were as follows: *GAPDH* F: 5′–ATGGAAATCCCATCACCATCTT−3′; *GAPDH* R: 5′–CGCCCCACTTGATTTTGG−3′; *TNF* F: 5′–CCTCTCTCTAATCAGCCCTCTG−3′; *TNF* R: 5′–GAGGACCTGGGAGTAGATGAG−3′; *IL1B* F: 5′–ATGATGGCTTATTACAGTGGCAA−3′; *IL1B* R: 5′–GTCGGAGATTCGTAGCTGGA−3′; *MMP14* F: 5′–CCATTGGGCATCCAGAAGAGAGC−3′; *MMP14* R: 5′–GGATACCCAATGCCCATTGGCCA−3′.

### RNA-sequencing

After the sample's total RNA was extracted, mRNA was purified using magnetic beads and oligo (dT). The resulting mRNA was then mixed with fragmentation buffer to create small fragments. Six base random primers were then used to create the first strand of cDNA using the fragmented mRNA as a template, and buffer containing dNTPs, RNase H and DNA polymerase I were added to create the second strand. Base A and sequencing adapter Y were added to both ends of the produced double-stranded cDNA, and PCR amplification was used to create the complete library. Illumina HiSeq2000 was utilized for the sequencing and analysis of the library.

### Luciferase assay

HEK293T cells were transiently transfected with firefly luciferase reporter (200 ng), TK-Renilla luciferase reporter (10 ng), and other corresponding plasmids. After 24 h, the Dual-Luciferase Reporter Assay System (Abcam, ab-228530) was used for luciferase assays. The firefly luciferase activity was normalized according to Renilla luciferase to obtain the relative luciferase activity.

### Statistical analysis

Data are presented as mean ± standard deviation (SD). GraphPad Prism 8 was used for statistical analysis. The reproducibility of experiments and the statistical tests used are presented in the figure legends.

## Data availability

The mass spectrometry data have been uploaded to Proteomics IDEntifications Database (PRIDE) with reference numbers 1-20250409-122324-3621951 (for Syntenin-1 interactome) and 1 to 20250409 to 124256 to 3621951 (for identification of Syntenin-1 phosphorylation sites). RNA sequencing data from syntenin-1-KO A549 cells re-expressing WT/S6A/S6D syntenin-1 has been deposited in the NCBI Sequence Read Archive (SRA) repository under accession codes: BioProject ID PRJNA1248217.

## Supporting information

This article contains supporting information.

## Conflict of interest

The authors declare that they have no conflicts of interest with the contents of this article.
